# The Solution of Urinary Calculi in Dilute Saline Solutions, at the Temperature of the Body, by the Aid of Electricity

**Published:** 1853-03

**Authors:** H. Bence Jones

**Affiliations:** Physician to St. George’s Hospital


*The Solution of Urinary Calculi in Dilute Saline Solutions, at the Temperature of the Body, by the aid of Electricity.* By H. BENCE JONES, M. D., F. R. S., Physician to St. George's Hospital.—This paper contained the record of a number of experiments made to determine whether, out of the body, urinary calculi could be dissolved by placing them in dilute solutions of nitrate of potash and other salts, and then decomposing the solution in contact with the calculus by means of the galvanic battery. The urinary calculus was carefully dried and weighed, then fixed between the poles of a galvanic battery, after which it was immersed in a solution of nitre, and at the end of the experiment it was re-dried and re-weighed. The loss of weight gave the effect which was produced. The different calculi which had been used were also exhibited, showing the different degrees in which the various kinds of urinary calculi are dissolved when submitted to this treatment. The conclusions at which the author arrived, may be thus stated: In a solution of nitre containing 20 grains to the ounce, kept at the temperature of the body, uric acid calculi can be dissolved by the aid of electricity, at the rate of from 2 to 9 grains an hour. The solution takes place at the alkaline or negative pole. In the same time, and under the same circumstances, phosphatic calculi can be dissolved at the rate of from 2 to 25 grains. The solution takes place at the acid or positive pole. Calculi, consisting of oxalate of lime, proved to be far less soluble, usually not more than half a grain an hour, and at most 2 grains being dissolved. At the conclusion of the reading of the paper, the author stated, that he had been engaged in making further experiments with a solution of nitrate of potash containing only 10 grains to the ounce; and he exhibited some large uric acid and phosphatic calculi, which had been partially dissolved by the decomposition of this solution at the surface of the calculi. He also showed a catheter or litholyte, made by Weiss, which fulfilled the conditions requisite in an instrument for effecting the solution of urinary calculi in the body. It resembled an ordinary lithotrite, but the blades were—1st, isolated so as to conduct the electricity to the surface of the stone when it had been caught; 2ndly, the external surfaces of the blades were guarded, so that in case they came in contact with the mucous membrane, no chemical action would be there set up; 3rdly, a double channel for the injection of the solution of nitre was formed inside the instrument. Lastly, the author stated, that although many difficulties would have doubtless to be overcome before he could lay the result of his experiments within the body before the Society, still they would only be mechanical difficulties. The principle, which consisted in setting up mechanical action at the spot where it was wanted, whilst elsewhere a dilute neutral solution was present, left nothing further to be desired; at least so far as the solution of uric and phosphatic calculi was concerned. At present, by the aid of the lithotrite, mechanical force is applied to the surface of the calculus, and the stone is passed in fragments. At some future time, by the aid of the litholyte, chemical force will be set up at the surface of the calculus, and it will be passed in solution, or as an impalpable precipitate. —*London Med. Times.*

